# Supplementary figures and images for: pyTCR: A comprehensive and scalable solution for TCR-Seq data analysis to facilitate reproducibility and rigor of immunogenomics research
Source: Front Immunol. 2022 Oct 27;13:954078. doi: 10.3389/fimmu.2022.954078 (PMC9704496; doi:10.3389/fimmu.2022.954078)

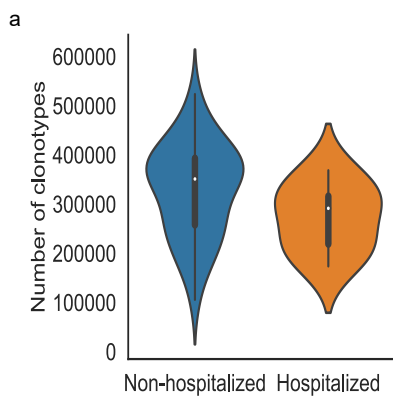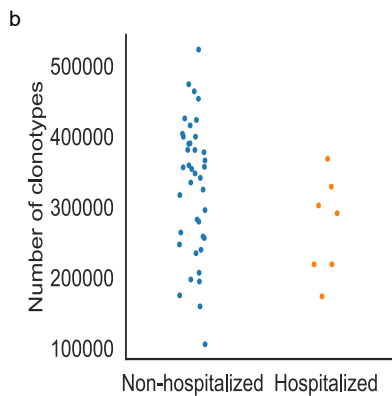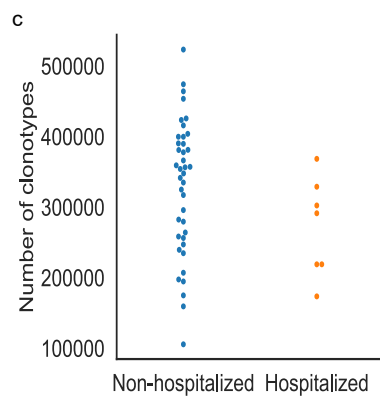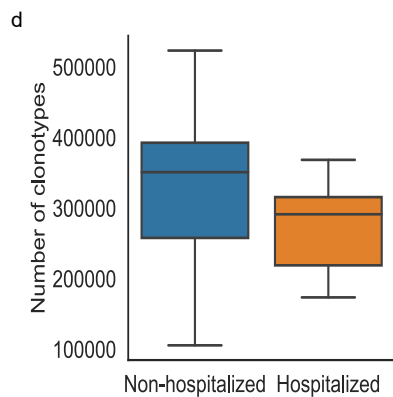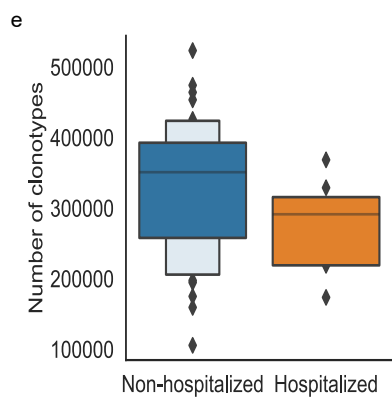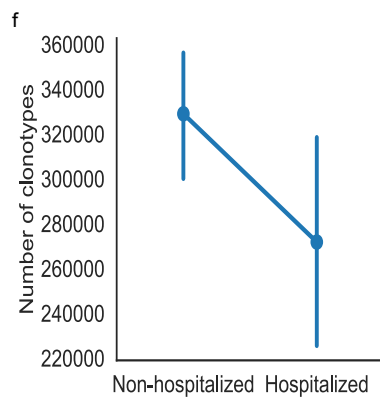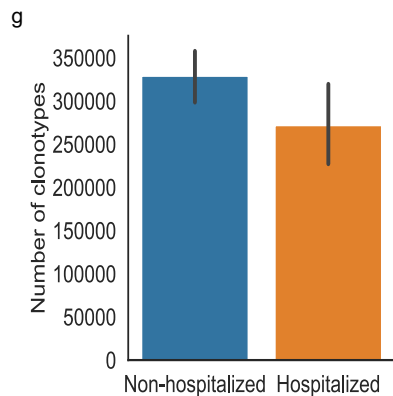

Supplement: Supplementary Figure 1 — The clonotype counts were shown in groups by hospitalization status. The patients that were not hospitalized were shown in blue while the patients that were hospitalized were shown in orange. Different types of plots were shown as follows: (A) violin plot (B) strip plot (C) swarm plot (D) box plot (E) boxen plot (F) point plot (G) bar plot. [file Image_1.pdf]

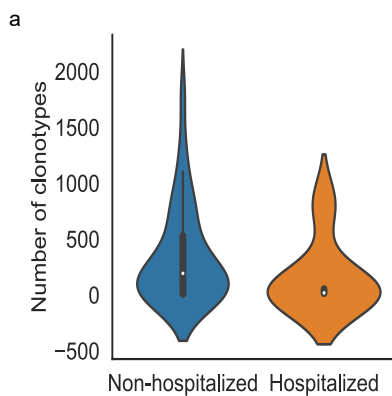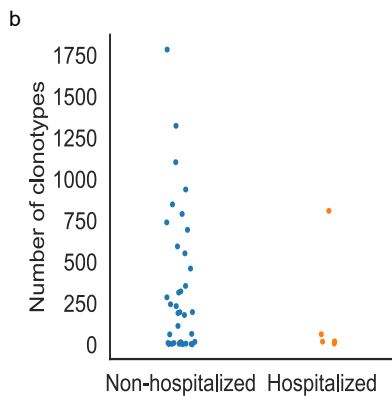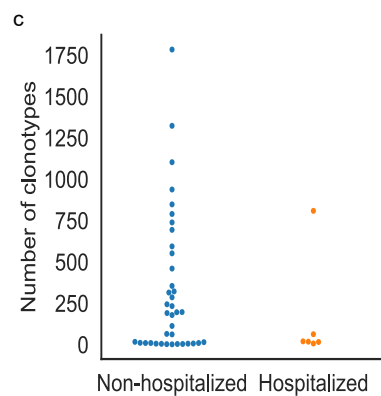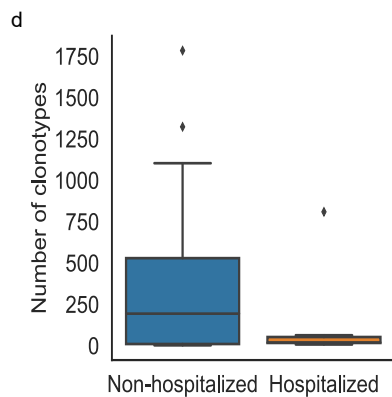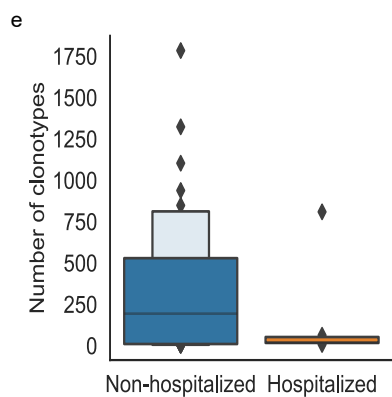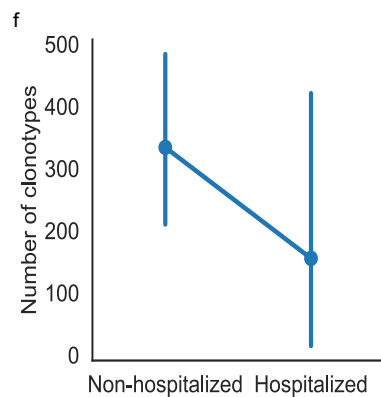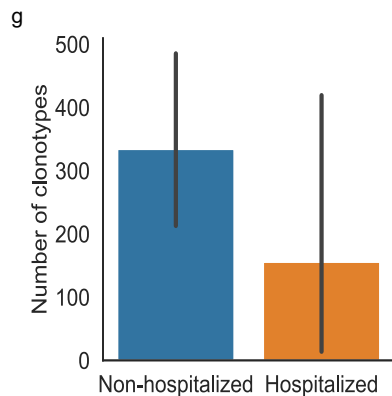

Supplement: Supplementary Figure 2 — Clonal portion grouped by hospitalization status. The y-axis presented the number of clonotypes that counted for 10% of all the clonotypes in the repertoire. The patients that were not hospitalized were shown in blue while the patients that were hospitalized were shown in orange. Different types of plots were shown as follows: (A) violin plot (B) strip plot (C) swarm plot (D) box plot (E) boxen plot (F) point plot (G) bar plot. [file Image_2.pdf]

a

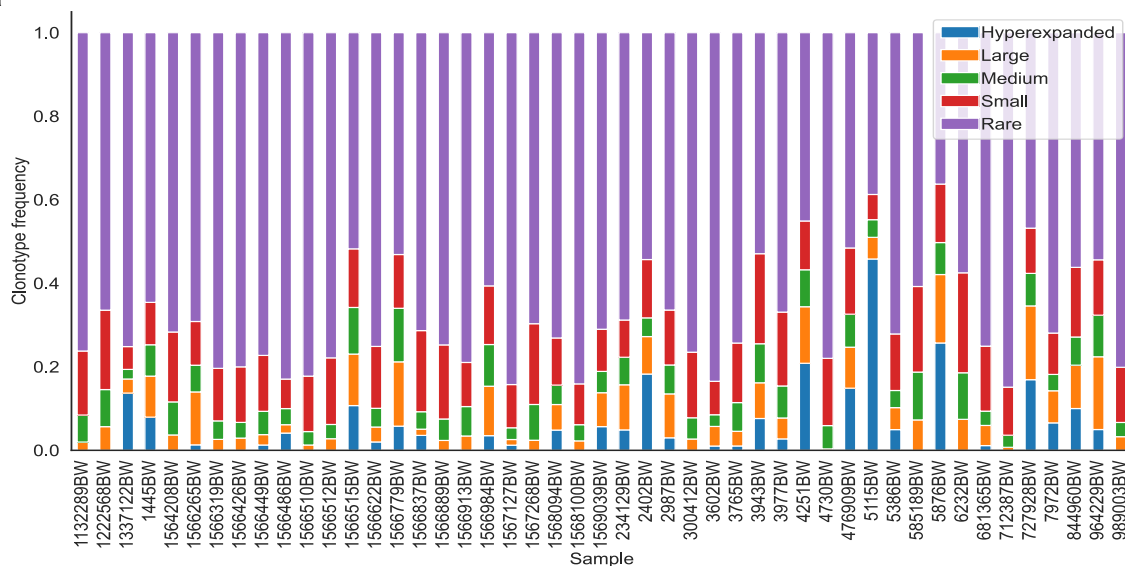

b

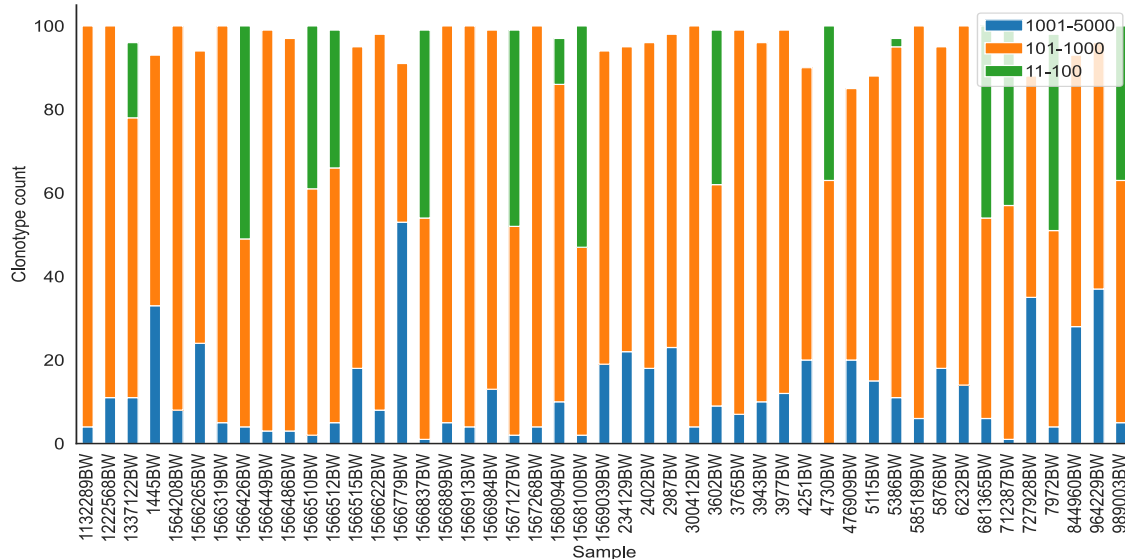

c

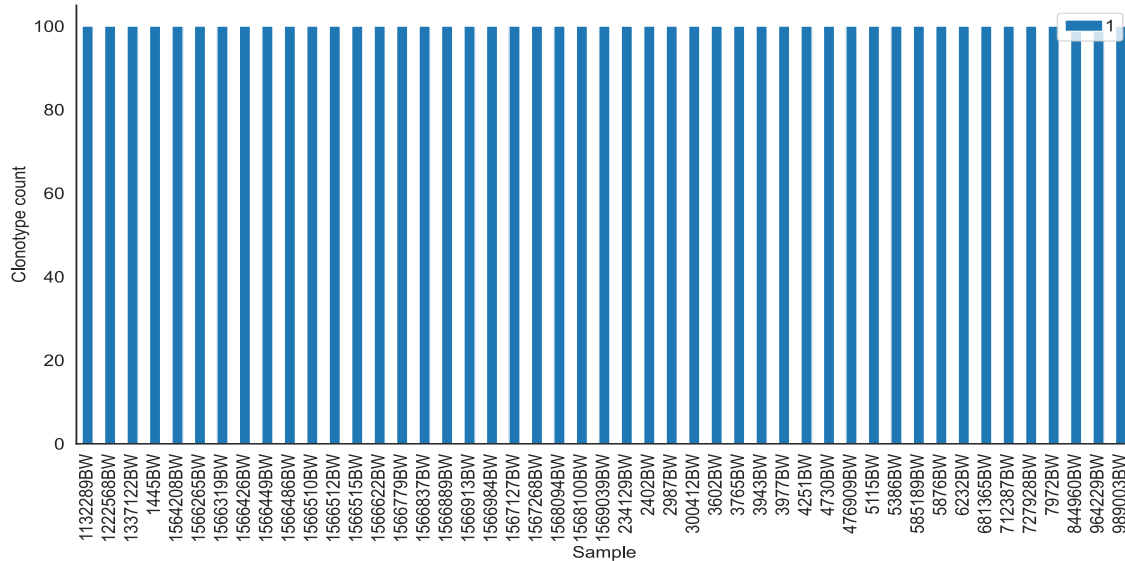

Supplement: Supplementary Figure 3 — The distribution of clonotype groups in each sample. (A) The distribution of clonotype groups was based on the clonotype frequency across all the clonotypes. Hyperexpanded clonotypes (blue) were the clones with frequencies between 0.01 to 1, large clonotypes (orange) were the clones with frequencies between 0.001 to 0.01, medium clonotypes (green) were the clones with frequencies between 0.0001 to 0.001, small clonotypes (red) were the clones with frequencies between 0.00001 to 0.0001, rare clonotypes (purple) were the clones with frequencies between 0 to 0.00001. (B) The distribution of clonotype groups based on the clonotype count across the top 100 clonotypes. The clonotypes with clone counts between 1001-5000 were presented in blue, the clonotypes with clone counts between 101-1000 were resented in orange, and the clonotypes with clone counts between 11-100 were presented in green. (C) The distribution of clonotype groups based on the clonotype count across the rare 100 clonotypes. The clonotypes with clone count of 1 were presented in blue. [file Image_3.pdf]

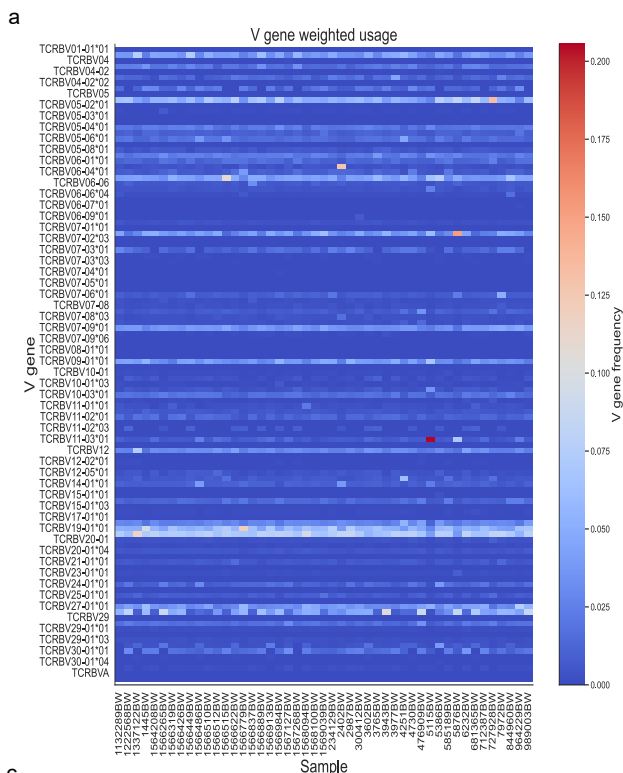

Supplement: Supplementary Figure 4 — Heatmap of the weighted V gene usage in each sample and the Sankey plot for V-J combinations. (A) The heatmap of V gene weighted usage (B) The hierarchically-clustered heatmap of V gene weighted usage. x-axis represented each sample, y-axis represented different V genes. The shade of the color corresponded to the V gene frequency. (C) The V-J combinations with V and J gene frequencies in sample 3602BW were presented as a Sankey plot. [file Image_4.pdf]

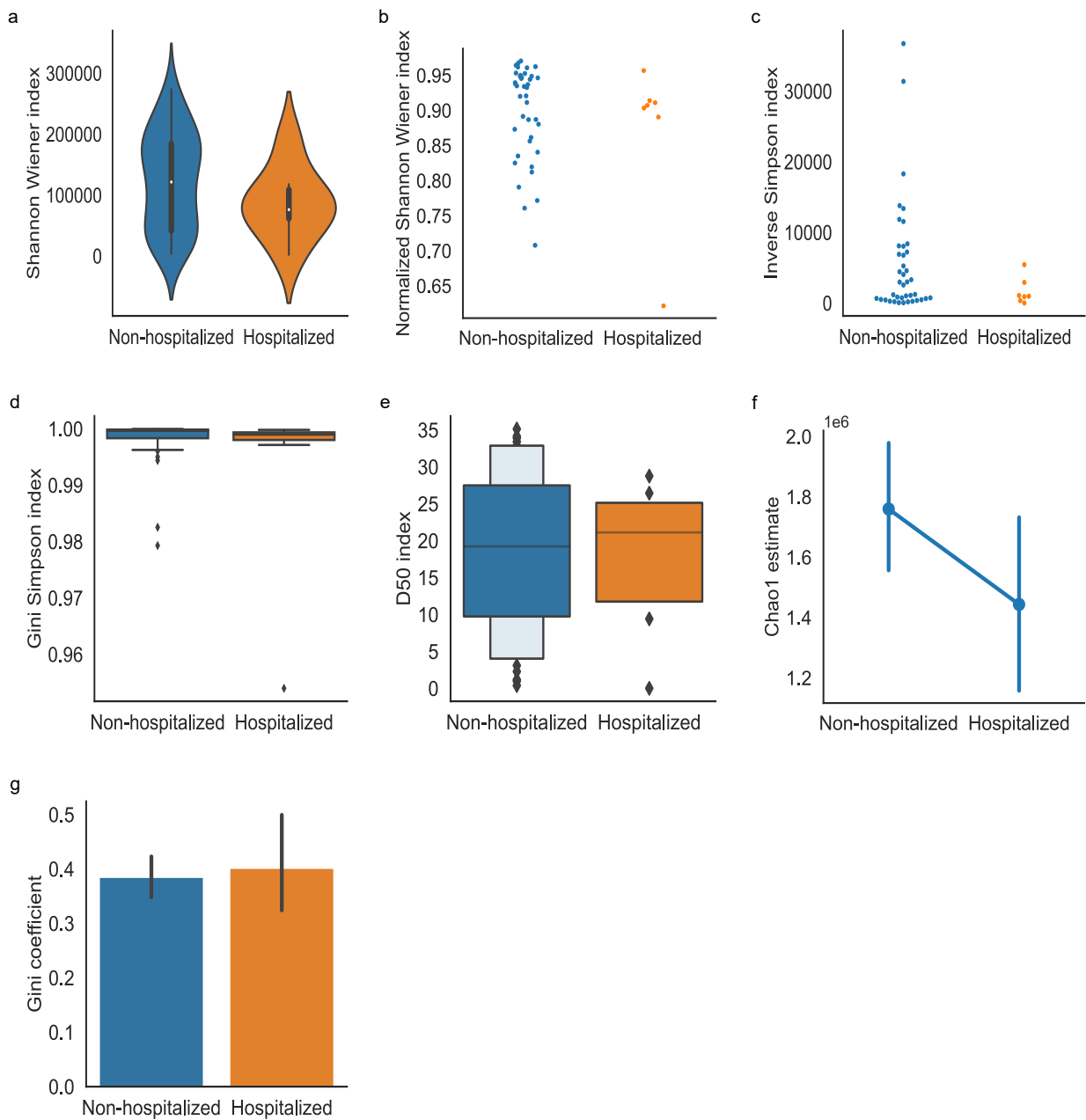

Supplement: Supplementary Figure 5 — The diversity indices were shown in groups by hospitalization status. The patients that were not hospitalized were shown in blue while the patients that were hospitalized were shown in orange. (A) violin plot of the Shannon Wiener index (B) strip plot of normalized Shannon Wiener index (C) swarm plot of inverse Simpson index (D) box plot of Gini Simpson index (E) boxen plot of D50 index (F) point plot of Chao1 estimate (G) bar plot of Gini coefficient. [file Image_5.pdf]

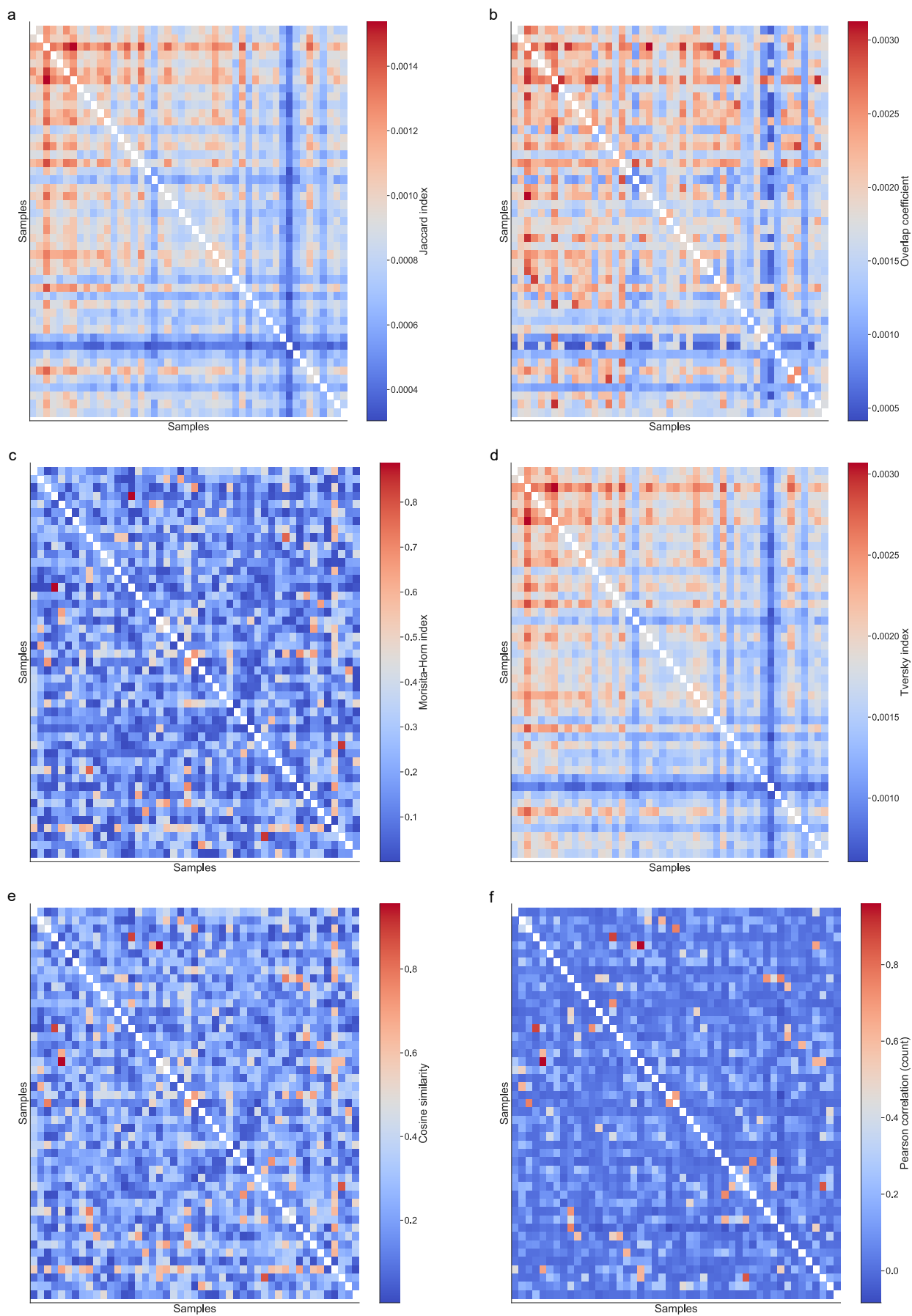

g

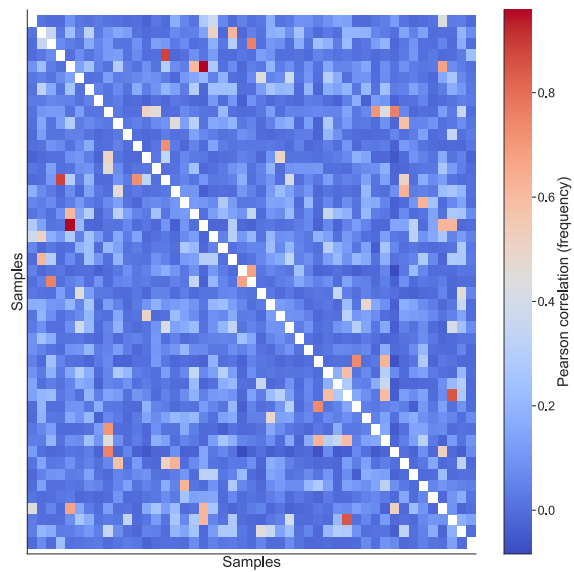

h

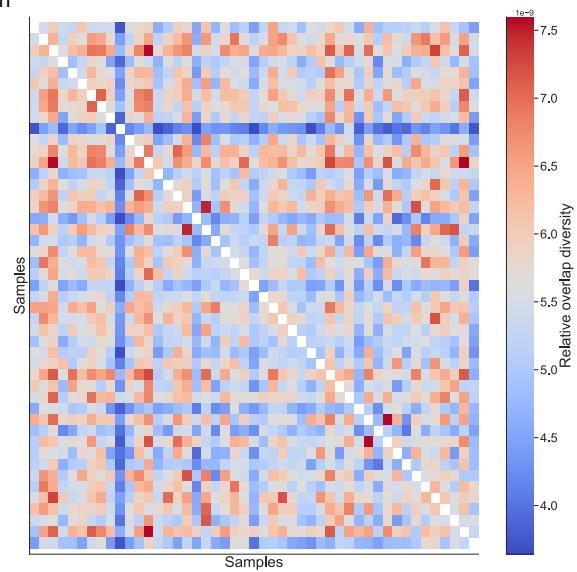

i

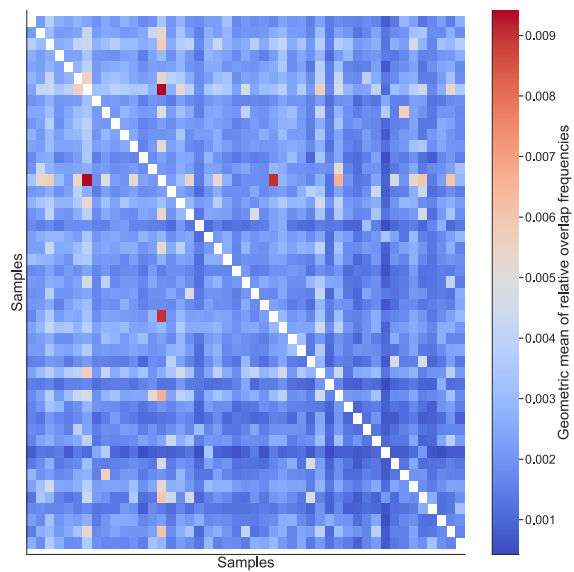

j

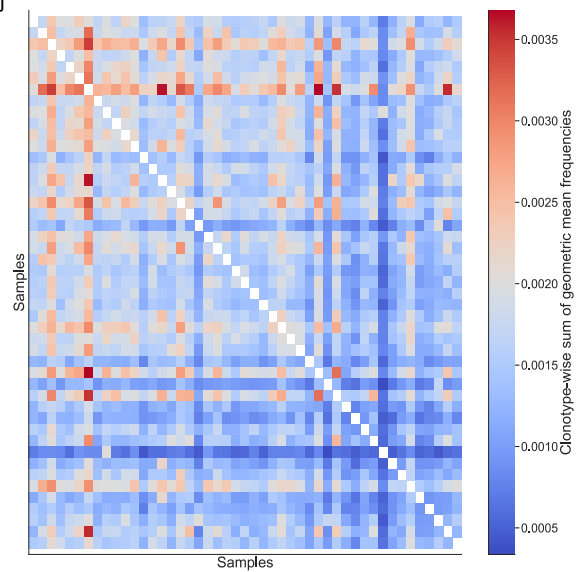

k

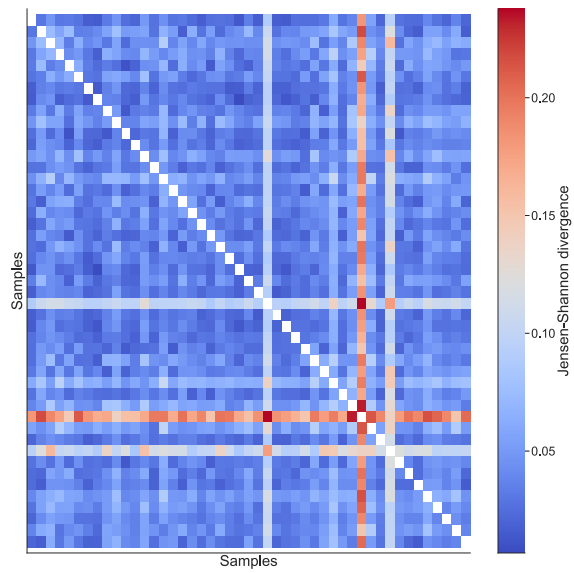

Supplement: Supplementary Figure 6 — The overlap indices across samples. Heatmaps of each overlap index as shown above. The x-axis and the y-axis presented each sample. (A) Jaccard index (B) Overlap coefficient (C) Morisita-Horn index (D) Tversky index (E) Cosine similarity (F) Pearson correlation based on clonotype counts (G) Pearson correlation based on clonotype frequency (H) Relative overlap diversity (I) Geometric mean of relative overlap frequencies (J) Сlonotype-wise sum of geometric mean frequencies (K) Jensen-Shannon divergence of variable gene usage distributions. [file Image_6.pdf]

a

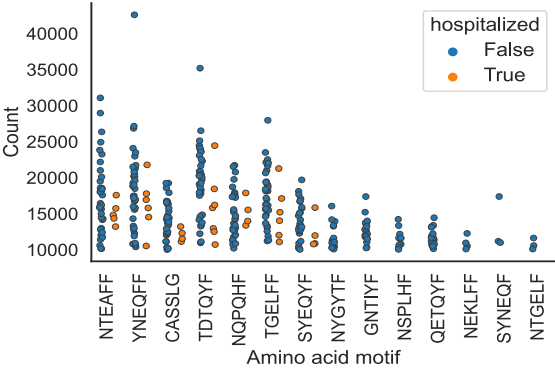

b

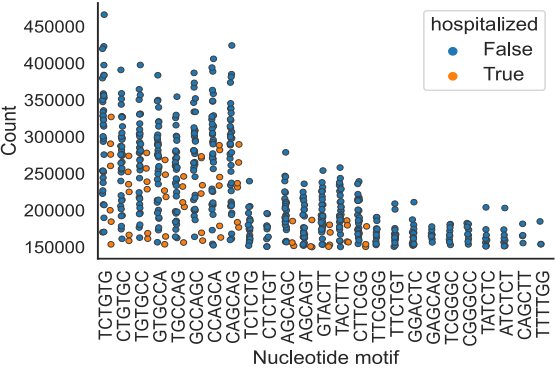

Supplement: Supplementary Figure 7 — The number of highly presented motifs across samples grouped by hospitalization status. The x-axis presented motifs and the y-axis presented the count of the motifs. (A) amino acid motifs (k=6) that had numbers of counts of no less than 9,999 and were presented in more than 2 samples in hospitalization and non-hospitalization groups were shown in the strip plot (B) nucleotide motifs (k=6) that had numbers of counts of more than 150,000 and were presented in more than 2 samples in hospitalization and non-hospitalization group were shown in the strip plot. [file Image_7.pdf]
