# Supplementary material for: pyTCR: A comprehensive and scalable solution for TCR-Seq data analysis to facilitate reproducibility and rigor of immunogenomics research
Source: Front Immunol. 2022 Oct 27;13:954078. doi: 10.3389/fimmu.2022.954078 (PMC9704496; doi:10.3389/fimmu.2022.954078)
Supplement: Supplementary file 8 [file Table_1.docx]

**Supplementary Table**

Supplementary Table 1. The comparison of TCR repertoire analysis tools.

| Metric | pyTCR | VDJtools | Immunarch | VisTCR |
| --- | --- | --- | --- | --- |
| Basic analysis | | | | |
| Read count | Yes | Yes | Yes | No |
| Clonotype count | Yes | Yes | Yes | No |
| Mean frequency | Yes | Yes | No | No |
| Geometric mean frequency | Yes | Yes | No | No |
| Mean length of CDR3 nucleotide sequence | Yes | Yes | No | No |
| Convergence | Yes | Yes | No | Yes |
| Spectratype | Yes | Yes | Yes | Yes |
| Clonality analysis | | | | |
| The most or the least frequent clonotype | Yes | Yes | Yes | Yes |
| 1-Pielou index | Yes | No | No | No |
| Clonal proportion | Yes | No | Yes | No |
| Relative abundance (in all repertoire, top clonotypes, rare clonotypes) | Yes | No | Yes | No |
| Diversity analysis | | | | |
| Shannon-Wiener index | Yes | Yes | Presented by Hill numbers | Yes |
| Normalized Shannon-Wiener index | Yes | Yes | Presented by Hill numbers | No |
| Inverse Simpson index | Yes | Yes | Yes | Yes |
| Gini Simpson index | Yes | No | Yes | Yes |
| D50 index | Yes | Yes | Yes | No |
| Chao1 estimate | Yes | Yes | Yes | No |
| Gini coefficient | Yes | No | Yes | No |
| Gene usage analysis | | | | |
| V,D,J gene weighted usage | Yes | Yes | Yes | Yes |
| V,D,J gene unweighted usage | Yes | Yes | No | No |
| Overlap analysis | | | | |
| Morisita-Horn index | Yes | Yes | Yes | Yes |
| Jaccard index | Yes | Yes | Yes | Yes |
| Overlap coefficient | Yes | No | Yes | No |
| Tversky index | Yes | No | Yes | No |
| Cosine similarity | Yes | No | Yes | No |
| Pearson correlation  of clonotype frequencies | Yes | Yes | No | No |
| Relative overlap diversity | Yes | Yes | No | No |
| Geometric mean of relative overlap frequencies | Yes | Yes | No | No |
| Сlonotype-wise sum of geometric mean frequencies | Yes | Yes | No | No |
| Jensen-Shannon divergence | Yes | Yes | No | No |
| Motif analysis | | | | |
| Amino acid spectratype | Yes | No | Yes | No |
| Amino acid motif analysis | Yes | No | Yes | No |
| Nucleotide sequence motif analysis | Yes | No | No | No |
| Statistical analysis | | | | |
| Student's t-test | Yes | No | No | Yes |
| Wilcoxon rank-sum test | Yes | No | No | No |
| Bonferroni correction | Yes | No | No | No |
